# Supplementary material for: Design, Spectral Characteristics, Photostability, and Possibilities for Practical Application of BODIPY FL-Labeled Thioterpenoid
Source: Bioengineering (Basel). 2022 May 12;9(5):210. doi: 10.3390/bioengineering9050210 (PMC9138141; doi:10.3390/bioengineering9050210)
Supplement: Supplementary file 1 [file bioengineering-09-00210-s001.zip › bioengineering-1710061-supplementary.pdf]

Supplementary material

Contents.

N		Page
**1**	^{1}H NMR spectrum	S2
**2**	^{13}C-{^{1}H} NMR spectra	S3
**3**	500-MHz proton NMR spectra	S3
**4**	Table S1	S4
**5**	Table S2	S4
**6**	Table S3	S5
**7**	Table S4	S5

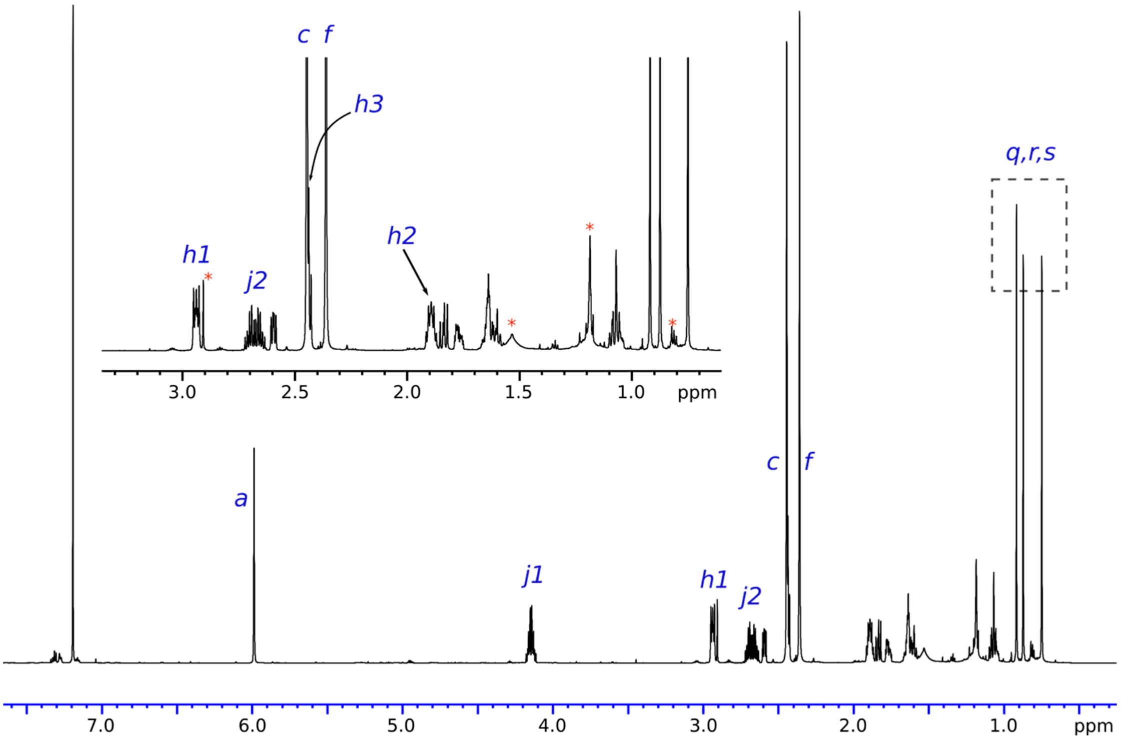

Figure S1. ^{1}H NMR spectrum (700 MHz) of terpene conjugate **6** in $CDCl_3$. Temperature t = 25°C, reference signal of chloroform is set to δ = 7.24 ppm. Signal labeling is as in Figure. S2 (or Figure 3 in the article text). Impurities are marked by asterisks.

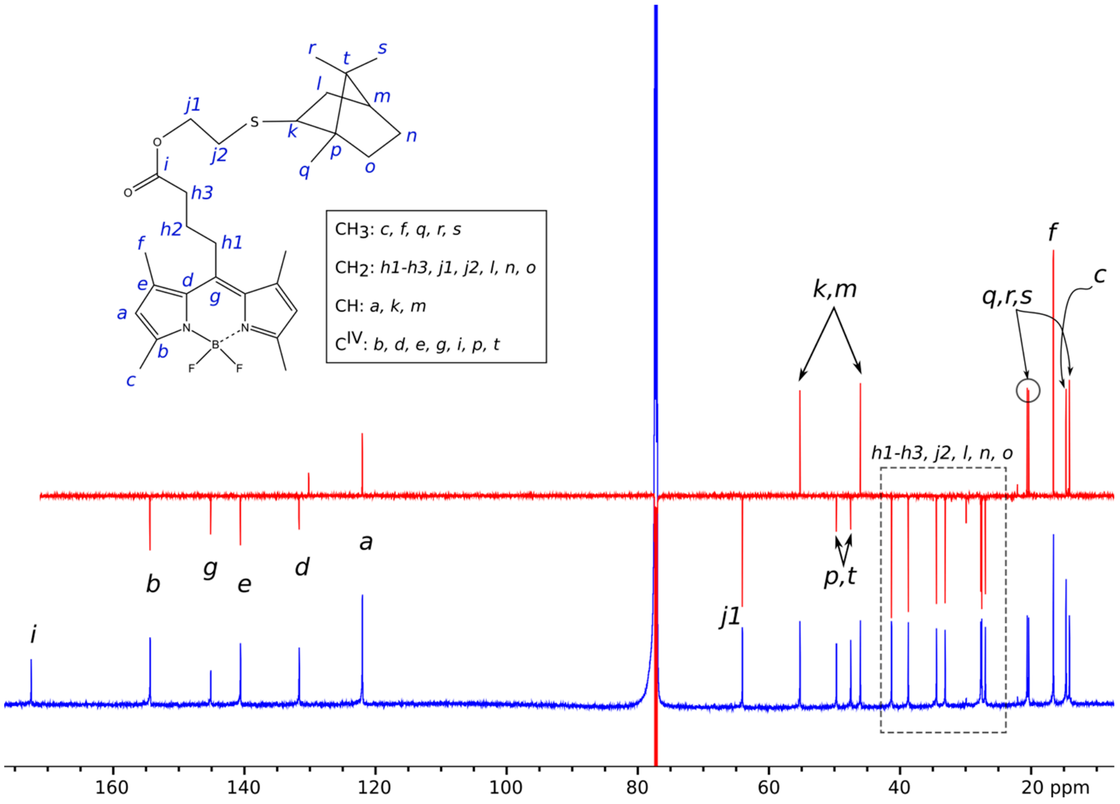

Figure S2. ^{13}C-{^{1}H} NMR spectra (176 MHz) of terpene conjugate **6** in $CDCl_3$. Temperature t = 25°C, reference signal of chloroform is set to δ = 77.23 ppm. Signal labeling is as in Figure 3 in the article text. The upper spectrum is the APT: positive lines belong to CH_3 and CH groups; negative, to CH_2 and quaternary carbons.

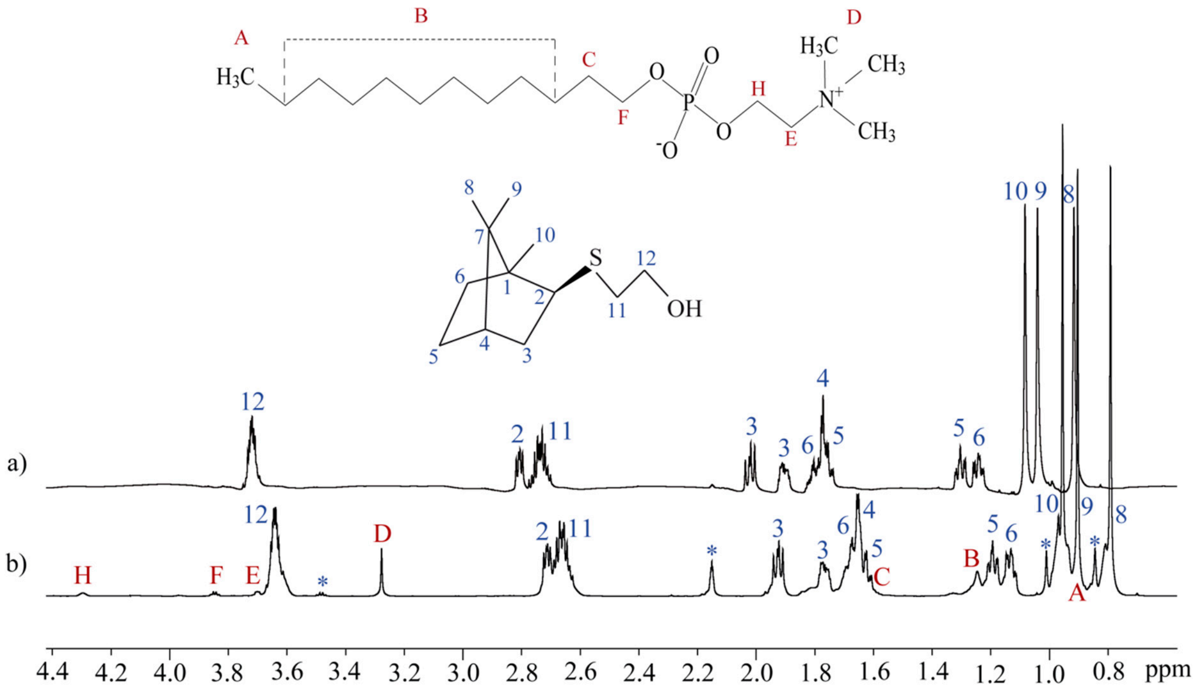

Figure S3. 500-MHz proton NMR spectra of bornane sulfide **5** in (a) $(CD_3)_2CO$ and (b) $(CD_3)_2CO+D_2O+DPC$ solutions. The impurity signals are marked by asterisks.

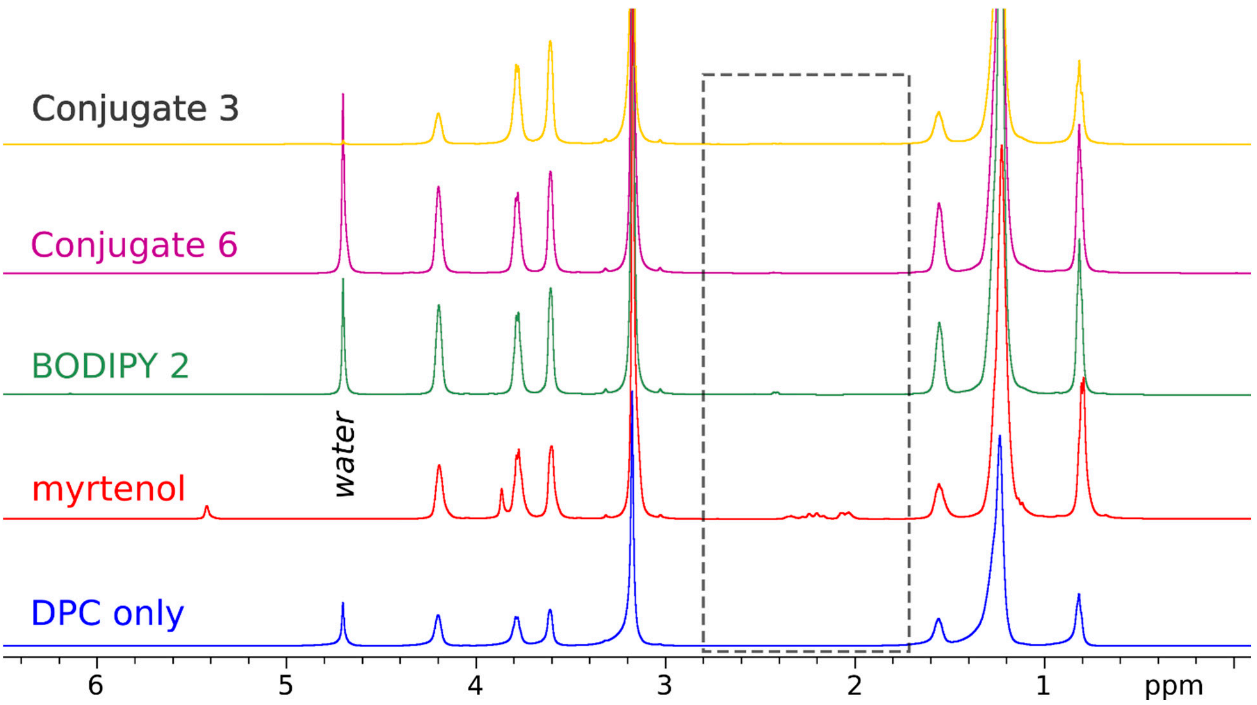

Figure S4. 1H NMR spectra (500.1 MHz, D2O) of the studied compounds in the presence of DPC micelles. From top: **3**, **6**, **2**, myrtenol, DPC. The zoomed region containing characteristic signals is marked with a rectangle and shown in Fig. 13.

Table S1. Torsion angles for two independent molecules of compound 6 in a crystal according to XRD data.

Angle	Molecule A, °	Molecule B, °
C2C3C17C18	-83.5(4)	-92.4(4)
C3C17C18C19	-179.6(3)	-178.5(3)
C17C18C19C20	69.4(4)	-66.8(4)
C18C19C20O21	-115.7(4)	117.7(4)
C19C20O21C22	177.7(3)	-173.7(3)
C20O21C22C23	176.0(4)	-173.4(3)
O21C22C23S24	-179.8(3)	178.0(3)
C22C23S24C25	-134.1(4)	-175.9(3)
C23S24C25C30	160.3(3)	155.3(3)

Table S2. Quantitative kinetic characteristics of the processes of photodegradation of dyes **2**, **6** and **7** in organic solvents.

**Compound**	**Solvent**							
	**Cyclohexane**		**1-Octanol**		**Toluene**		**DMSO**	
	$t_{1/2}$, **h**	$k_{obs}\cdot10^6$, s^{-1}	$t_{1/2}$, **h**	$k_{obs}\cdot10^6$, s^{-1}	$t_{1/2}$, **h**	$k_{obs}\cdot10^6$, s^{-1}	$t_{1/2}$, **h**	$k_{obs}\cdot10^6$, s^{-1}
**2**	88.6	2.9±0.3	82.3	3.1±0.3	40.5	5.5±0.3	8.3	27.7±0.3

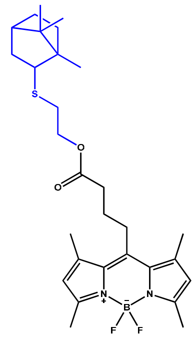 **6**	85.1	3.1±0.3	81.9	3.3±0.2	40.4	5.6±0.2	8.1	28.1±0.3
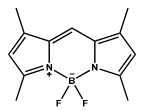 **7**	46.0	4.8±0.3	30.4	6.9±0.5	16.7	8.8±0.6	4.7	41.2±0.5

Table S3. ^{1}H NMR chemical shifts (δ, ppm) of bornane sulfide **5** in $(CD_3)_2CO$ and $(CD_3)_2CO+D_2O$+DPC solutions.

**Solvent**	**CH-2**	**CH_2-3**	**CH-4**	**CH_2-5**	**CH_2-6**	**CH_3-8**	**CH_3-9**	**CH_3-10**	**CH_2-11**	**CH_2-12**
$(CD_3)_2CO$	2.81	1.90, 2.02	1.77	1.30, 1.75	1.24, 1.81	0.92	1.04	1.08	2.73	3.72
$(CD_3)_2CO$ + D_2O + DPC	2.71	1.77, 1.92	1.65	1.19, 1.62	1.13, 1.68	0.79	0.90	0.95	2.66	3.64

Table S4. ^{13}C NMR chemical lshifts (δ, ppm) of bornane sulfide **5** in $(CD_3)_2CO$ and $(CD_3)_2CO+D_2O$+DPC solutions.

Solvent	C-1	CH-2	CH_2-3	CH-4	CH_2-5	CH_2-6	C-7	CH_3-8	CH_3-9	CH_3-10	CH_2-11	CH_2-12
$(CD_3)_2CO$	47.1	54.5	41.2	45.9	38.3	27.2	49.1	19.9	20.2	13.7	37.1	61.6
$(CD_3)_2CO$+ D_2O + DPC	46.9, 47.0′	54.4, 54.5′	40.8, 41.1′	45.7, 45.8′	38.0, 38.2′	26.9, 27.1′	49.0, 49.1′	19.7, 19.9′	20.0, 20.2′	13.5, 13.7′	36.2, 36.7′	61.1, 61.3′
